# Overview of “Systematic Reviews” of the Built Environment's Effects on Mental Health

**DOI:** 10.1155/2020/9523127

**Published:** 2020-03-19

**Authors:** Solange Núñez-González, J. Andrés Delgado-Ron, Christopher Gault, Adriana Lara-Vinueza, Denisse Calle-Celi, Riccardo Porreca, Daniel Simancas-Racines

**Affiliations:** ^1^Centro Asociado Cochrane Ecuador, Facultad de Ciencias de la Salud Eugenio Espejo, Universidad UTE, Quito 170129, Ecuador; ^2^Centro de Investigación en Salud Pública y Epidemiología Clínica (CISPEC), Universidad UTE, Quito 170129, Ecuador; ^3^School of Population and Public Health, Faculty of Medicine, The University of British Columbia, Canada; ^4^Facultad de Arquitectura, Universidad UTE, Quito 170147, Ecuador

## Abstract

Good mental health is related to mental and psychological well-being, and there is growing interest in the potential role of the built environment on mental health, yet the evidence base underpinning the direct or indirect effects of the built environment is not fully clear. The aim of this overview is to assess the effect of the built environment on mental health-related outcomes. *Methods*. This study provides an overview of published systematic reviews (SRs) that assess the effect of the built environment on mental health. We reported the overview according to the Preferred Reporting Items for Systematic Reviews and Meta-Analyses (PRISMA) guidelines. Databases searched until November 2019 included the Cochrane Database of Systematic Reviews, EMBASE, MEDLINE (OVID 1946 to present), LILACS, and PsycINFO. Two authors independently selected reviews, extracted data, and assessed the methodological quality of included reviews using the Assessing Methodological Quality of Systematic Reviews-2 (AMSTAR-2). *Results*. In total, 357 records were identified from a structured search of five databases combined with the references of the included studies, and eleven SRs were included in the narrative synthesis. Outcomes included mental health and well-being, depression and stress, and psychological distress. According to AMSTAR-2 scores, the quality assessment of the included SRs was categorized as “high” in two SRs and as “critically low” in nine SRs. According to the conclusions of the SRs reported by the authors, only one SR reported a “beneficial” effect on mental health and well-being outcomes. *Conclusion*. There was insufficient evidence to make firm conclusions on the effects of built environment interventions on mental health outcomes (well-being, depression and stress, and psychological distress). The evidence collected reported high heterogeneity (outcomes and measures) and a moderate- to low-quality assessment among the included SRs.

## 1. Introduction

Mental health is the well-being of the individual and the sum of his/her abilities to contribute to the community and adequately handle the daily stages of stress [1]. According to the World Health Organization (WHO), one in four people will be affected by a mental health problem at some point in their lives [2]. Mental disorders impose an enormous global disease burden, affecting every community and age group across all income countries [3], and it accounts for an estimated 32.4% of years lived with disability (YLDs) and 13.0% of disability-adjusted life years (DALYs) [4].

The total economic output lost to these disorders was estimated to be one trillion USD per year due to lost production and consumption opportunities [5]. The global cost of mental disorders was estimated to be 2.5 trillion USD in 2010, and these costs could rise to six trillion USD by 2030 [6].

Mental health depends not only on individual characteristics but also on health determinants which require an analysis of the influence of the environment on the individual's ability to stay healthy [7, 8].

The built environment is a broad term that encompasses the man-made physical elements of the environment such as homes, buildings, streets, open spaces, and infrastructure, which could have an impact on the physical and mental levels of the person and the health of a community [9].

Research on the association between built environment and health has increased in recent years; Smith et al. reported that improving neighborhood walkability and quality of green areas and providing adequate active transport infrastructure are likely to generate positive impacts on activity in children and adults [10]; on the other hand, Nowak et al. reported that a poor-quality built environment is related to negative birth outcomes [11].

Studies about the relationship between built environments and mental health have reported that a state of well-being, response to stressors, the ability to work productively, and to make contributions to the community all can be affected by factors such as the quality of public utilities, walking distance to public spaces, access to transport, and level of infrastructure [8, 12–17]. Generaal et al. reported through analysis across eight Dutch cohort studies that urbanization is associated with depression, indicating that a wide range of environmental aspects may relate to poor mental health [18].

This overview aims to collect, summarize, critically assess, and interpret the evidence related to the systematic reviews (SRs) of the built environment on mental health.

## 2. Methods

We conducted an overview that evaluated the adequate process and quality of SRs of built environment interventions and their effects on mental health status. The protocol was registered in PROSPERO, an international prospective register of systematic review protocol (registration number: CRD42018102676), and we reported the overview according to the Preferred Reporting Items for Systematic Reviews and Meta-Analyses (PRISMA) guidelines [19] ([Supplementary-material supplementary-material-1]).

### 2.1. Inclusion and Exclusion Criteria

The overview included any systematic reviews (SRs) and meta-analyses that reported a structured quality evaluation of included studies, as well as SRs published in peer-reviewed, scientific journals. Inclusion criteria were organized following the patient, intervention/exposition, comparison, and outcome (PI/ECO) reporting structure. Participants (P) included the entire population (without restrictions). Intervention/exposition (I/E) included the built environment, as defined by the National Library of Medicine's controlled vocabulary thesaurus (MESH), described above [9]. Comparisons (C) were not specified for the purpose of the inclusion criteria of the overview of SRs, but comparators reported in the original SRs were considered in the analysis. Outcomes (O) included mental health, as defined by the National Library of Medicine's controlled vocabulary thesaurus (MESH), described above [1]. We excluded SRs that do not include primary studies and SRs with outcomes not related to mental health.

### 2.2. Search Strategy

The search strategy was designed to identify all existing published systematic reviews and meta-analyses. Search terms were formulated using the PICO structure. A systematic literature review was conducted by searching the Cochrane Database of Systematic Reviews, EMBASE, MEDLINE (OVID 1946 to present), LILACS, and PsycINFO, published in English and Spanish. We ran the most recent search on November 2019. We reviewed references of all included articles in order to identify additional studies.

The complete search strategy is shown in Supplementary Material S2. The search results were imported into Rayyan [20], an online tool that provides procedural support in the selection of articles for systematic reviews.

### 2.3. Study Selection and Data Extraction

Two authors independently screened all titles and abstracts of studies identified by the search strategy against inclusion and exclusion criteria, and when eligibility was determined, we read the full text. Discrepancies around inclusion were resolved by discussion or in consultation with a third author when required. We searched the reference lists of all included reviews to identify any further relevant reviews. Citations were downloaded and managed in Mendeley.

Two authors independently extracted data from each SR into a purpose-built, predesigned, structured template. The data extraction forms were then summarized in a table and reviewed independently by a third reviewer.

### 2.4. Assessment of Methodological Quality

Two reviewers independently assessed the methodological quality of the included SRs using the Assessing Methodological Quality of Systematic Reviews-2 tool (AMSTAR-2) [21]. Each question in the AMSTAR-2 tool is answered as either “yes,” “partial yes,” “no,” “can't answer,” or “unable to assess”. Overall confidence in the quality rating in the SRs was classified as high, moderate, low, or critically low depending on the presence of critical and noncritical flaws in items 2, 4, 7, 9, 11, 13, and 15 [21]. We resolved any disagreements via a consensus decision by a third reviewer. Interobserver agreement was assessed with the kappa coefficient for each item and the total AMSTAR-2 score.

We categorized conclusions reported by authors for each SR, into six categories: “inconclusive,” “no effect,” “probably harmful,” “harmful,” “probably beneficial,” and “beneficial” (see Table 1 for further details of the category definition). Two reviewers independently categorized the conclusions. Discrepancies were discussed until consensus was reached. In all cases, judgement represented a formal assessment about the evidence, benefits, and harms of each intervention.

### 2.5. Data Analysis and Narrative Synthesis

We presented our findings through tables to describe the characteristics of the included SRs. The outcome components were listed, followed by a narrative synthesis that included understanding components of the interventions, exploring patterns of findings across studies, and giving greater weight to studies of higher quality in the interpretation of the findings, especially if there were contradictions between the findings of reviews.

Additionally, to analyze the overlap of included SRs, we used a citation matrix that crosslinks the SRs with their included primary studies to calculate the “corrected covered area” (CCA). Based on the reported of CCA value, we classified into four categories: slight (0–5%), moderate (6–10%), high (11–15%), and very high (>15%) overlap [22].

## 3. Results

### 3.1. Literature Search Results

**Figure 1:**
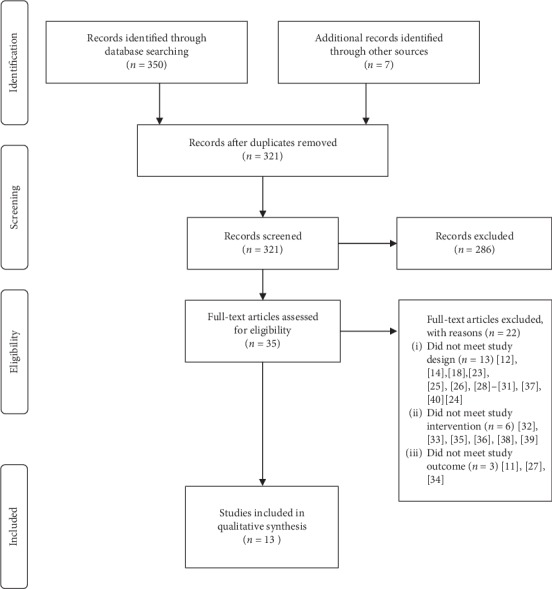
Flow diagram of the literature search and study selection.

We identified 350 records from the search strategies updated until November 2019 and seven more from the references of the included studies. After removing duplicates, 321 were manually screened, and 286 records were excluded for title and abstract. We reviewed the full text of 35 studies, 24 of which were excluded [11, 12, 14, 18, 23–42]. Finally, eleven SRs were included in the narrative synthesis (Figure 1).

### 3.2. Characteristics and Quality of the Systematic Reviews

Included SRs were published between 2010 and 2019, and they comprised studies conducted between 1991 and 2017. The last search was conducted in September 2017 [43]. One out of eleven included SRs performed a meta-analysis of data.  Table 2 describes the main characteristics of the included studies.

The quality of the included SRs according to AMSTAR-2 scores was categorized as “high” in two SRs [49, 50] and as “critically low” in nine SRs [15, 43–48, 51, 52] (Table 3). Drawbacks in the critical items included: the SRs did not state prior design or registered protocol [15, 43, 45–48, 51, 52], did not include list of excluded studies with reasons [15, 43–48, 51, 52], and did not address the risk of bias in the individual studies [15, 43–45, 47, 52]. Drawbacks in the noncritical items included: the authors did not report on the sources of funding for the studies included in the SRs [15, 43–52], and the SRs did not report conflicts of interest [43, 47]. The kappa coefficient for the total AMSTAR-2 score showed substantial agreement (0.78; 95% confidence interval (CI) 0.67–0.88).

#### 3.2.1. Mental Health and Well-Being

Bowler et al.'s review found that natural environments, when compared to synthetic environments, might have direct and positive impacts on well-being such as anger (Hedges' *g* = 0.46; 95% CI = 0.23, 0.69), fatigue (Hedges' *g* = 0.42; 95% CI = 0.07, 0.76), and sadness (Hedges' *g* = 0.36; 95% CI = 0.08, 0.63) [44]. Gascon 2017 et al.'s review suggested a positive association between exposure to outdoor blue spaces and mental health and well-being; however, the evidence of any direct causation was limited [46].

Ige et al.'s review suggests that affordable housing of good quality, with good energy efficiency and adequate ventilation, has the potential to be an important contributor to improved well-being [48]. Friesinger et al.'s review indicated that well-being was more likely to be linked to community and neighbourhood qualities than to a specific building, while deterioration in the physical quality of the neighbourhood exacerbated mental health problems [43].

van den Berg et al.'s review found that adults who live in green neighbourhoods report better mental health than adults who live in less green neighbourhoods, especially in population groups with lower socioeconomic status [51]. Zhang et al.'s review reported that people with mobility disabilities could gain mental health benefits and social health benefits from nature in different kinds of nature contacts ranging from passive contact, active involvement to rehabilitative interventions [52].

Gascon 2015 et al.'s review found limited evidence of the benefits of long-term residential surrounding greenness and mental health in adults, whereas the evidence was inadequate in children. Finally, Moore et al.'s review reported that the evidence for the impact of built environment interventions on mental health and well-being is weak, as the primary studies reported a very small or no effect of the built environment on mental health and well-being [45].

The CCA of 2.5% indicates a slight overlap of primary studies between the different SRs.

#### 3.2.2. Depression and Stress

Rautio et al.'s review reported that poor housing quality and nonfunctioning, lack of green areas, and noise and air pollution are more clearly related to depressive mood.

Turley et al.'s review included only one study which observed fewer symptoms of maternal depression and maternal stress in intervention households provided with cement floors as part of the “Piso Firme” project; however, there was no evidence available to assess the impact of slum upgrading on depression and stress.

The CCA of 0% indicates a slight overlap of primary studies between the different SRs.

#### 3.2.3. Psychological Distress

Gong et al.'s review suggested that some aspects of the urban environment including housing with deck access, neighbourhood quality, the amount of green space, land-use mix, industry activity, and traffic volume have significant associations with psychological distress.

According to the conclusions of the SRs reported by the authors, only one SR reported a “beneficial” effect on mental health and well-being outcomes (Table 4).

## 4. Discussion

Our overview collected information from eleven SRs published between 2010 and 2019 and included 178 primary studies (63% of them were cross-sectional). Eight SRs evaluated the mental health and well-being, and only one SR reported “high-quality” assessment; however, the authors concluded that the interventions had “no effect” [49]; two SRs evaluated depression and stress and one SR reported “high-quality” assessment; the authors concluded that the interventions would be “unclear” effect [50]; only one SR evaluated psychological distress and reported “critically low-quality” assessment, and the authors concluded that the effect of the interventions would be “probably beneficial” effect [47].

We found a slight overlap (2.5%) of primary studies between the different SRs looking at the outcomes of mental health/well-being and depression/stress. Only one of the included systematic reviews performed a meta-analysis [44], heterogeneity due to the design of primary studies, weak methodological rigor, and the lack of standardized tools that assess mental health, and built environments are the main drawbacks reported by the others SRs.

The research looking at the role of the built environment on mental health is relatively new, and causal pathways connecting both constructs are just starting to emerge. According to van den Bosch et al., mental health is consistently influenced directly or indirectly by multiple environmental exposures, and depressive mood may be the result of the rapid urbanization and a disconnection from our evolutionary origin and natural environments [53]. Frank et al. emphasize transportation infrastructure, land use, the pedestrian environment, and greenspace influence people's behavior and exposures [54]. Some of these, such as social cohesion, are directly related to mental health. While others may have an indirect effect, past research has suggested that green space could modulate the impact of stressors [55]. Similarly, the built environment plays a role in the perception of safety and the enjoyment of aesthetics and, as such, can impact the individual's mental health [56].

The high number of primary studies in the last 10 years reflects the increasing interest by the researchers and public health professionals in this area; however, a major limitation of this evidence is more than 50% of the included studies were cross-sectional; in these studies, the temporal relationship between exposure and outcome cannot be established [51]; therefore, it is not possible to conclude the causality of the built environment on mental health [47]. Hence, as mentioned in some SRs [48, 49, 52], there is a need for more high-quality research, especially controlled longitudinal/time-series analyses.

Regarding the effect assessment postintervention, we could not possibly assess whether the short- or long-term effects of the interventions are maintained, continue to improve, or worsen over time because none of the reviews reported this information.

To our knowledge, this is the first overview of systematic reviews rigorously looking at the relationship between the built environment and mental health. Clark and colleagues sought to do so in 2007; their review included mostly primary research studies—only three 3 out of 99 reviewed references were systematic reviews—leading them to conclude a “lack of robust research, and of longitudinal [studies] in many areas” [49]. Nearly thirteen years after they ran their search, systematic reviews have gone from three to eleven, integrating new specific outcomes such as depression, anxiety, and psychological distress. However, heterogeneity is still a major issue when assessing the impact of built environments on mental health [57].

Two government-sponsored scoping reviews from Canada and the UK were published in 2013 and 2018, respectively [58, 59]. The former studies the impact of housing circumstances and housing interventions in mental health while the latter explores the impact of natural environments. Because Johnson's review relies on evidence “relevant to public policy considerations and to the UK context,” his findings cannot easily be applied to the general population. Coghill et al., on the other hand, used a systematic approach to evidence synthesis but chose to focus exclusively on evidence related to natural environments published in 2012 or later.

While their findings are similar to ours, they chose a positive framing when writing their final conclusions and recommendations. On a final note, their review signaled several limitations that were identified by our team, making evident the need for an interdisciplinary approach similar to that put forward by Weaver and colleagues for physical health.

Interdisciplinary research between urban planning, architecture, psychology, environmental health, epidemiology, and sociology is crucial to fully grasp the pathways from the built environment to mental health [25]. The most salient dimensions of the built environment and contextual factors should be incorporated into more ecologically valid models [60].

### 4.1. Strengths and Limitations of the Overview

This overview evaluated the available evidence of the mental health outcome, which is considered a priority in public health, and this makes our overview relevant to current policymakers and stakeholders to adequately develop strategies to strengthen health promotion policies. We followed a rigorous systematic review method and reported according to PRISMA guidelines [19]. We did not limit our search by publication year; also, to limit bias, data selection, extraction, and assessment of methodological quality were performed by two people independently. The interrater agreement was excellent. Limitations of this overview include the use of a search filter limited to only systematic review matches, as well as the searches being limited to publications in English and Spanish, and this may have resulted in some studies being missed. We were also limited by the broad scope of our exposure, “the built environment” can range from small interventions indoors to big urban expansions outdoors in both urban and rural settings. As such, we did not aim to identify specific interventions but to measure the overall quality of the existing literature, as appraised by other authors. Finally, we could not account for mediators between the built environment and health such as noise, physical activity, social cohesion, temperature, or air pollution as this was outside the scope of our overview.

## 5. Conclusions

There was insufficient evidence available to make firm conclusions on the effects of built environment interventions on mental health outcomes (well-being, depression and stress, and psychological distress). The evidence collected reported high heterogeneity (outcomes and measures) and the high- to critically low-quality assessment. Future research efforts in the field should focus on improved methodological design to reduce the risk of bias and improved reporting through standardized tools that evaluate the different interventions and outcomes, as well as interdisciplinary research involving professionals specialized in mental health, public health, spatial planners, and urban design experts.

## Figures and Tables

**Table 1:** Classification of the conclusions according to the results reported by authors.

Classification	Definition
Unclear	The direction of results differed within reviews due to conflicting results or limitations of individual studies
No effect	The conclusions provided evidence of no difference between intervention and comparator
Probably harmful	The conclusions did not claim for firm harmful effect despite the reported negative treatment effect
Harmful	The conclusions were reported as clearly indicative of a harmful effect
Probably beneficial	The conclusions did not claim for firm benefits despite the reported positive treatment effect
Beneficial	The conclusions reported a clear beneficial effect without major concerns regarding the supporting evidence

**Table 2:** Characteristics of the included systematic reviews.

Author (Year)	Population/stage	Number of included studies^*∗*^	Interventions	Outcome	Meta-analysis	Quality assessment
Bowler et al. 2010 [44]	All ages	11studies (6 crossover trial, 1 observational study, 4 pretest-posttest comparison groups-randomised)	Exposure to natural environments: (i) Public parks (ii) Green university campuses	Well-being (i) Anger (ii) Fatigue (iii) Sadness (iv) Anxiety (v) Anger	Yes	Methodology quality checklist was devised by the authors
Friesinger et al. 2019 [43]	People with mental health problems	11 studies (6 cross-sectional, 1 longitudinal, 1mixed-method, 1participant observation-free analysis, 1 interview content analysis, and 1 photo-elicitation and interviews)	Housing type	Well-being	No	Critical Appraisal Skills Programme (CASP, 2017)
Gascon et al.2015 [45]	General population—all ages	28 studies (21 cross-sectional, 6 longitudinal, and 1 ecological study)	Residential green and blue spaces	Mental health	No	Quality score was based on 11 different items
Gascon et al.2017 [46]	General population—all ages	12 studies (7 cross-sectional, 4 longitudinal, and 1 pre/postobservational study)	Outdoor blue spaces	Mental health and well-being	No	Quality score was based on 11 different items
Gong et al 2016 [47]	General population—all ages	11 studies (11 cross-sectional)	Urban environment: (i) Architectural design (ii) Land use (iii) Walkability, connectivity, and accessibility (iv) Neighborhood and housing quality	Psychological distress (i) Depression (ii) Anxiety	No	Critical appraisal proforma developed and validated by the Health Evidence Bulletin Wales project
Ige et al.2018 [48]	General population—all ages	6 studies (1 randomised controlled trial, 1 quasiexperimental study, 1 before-and-after studies, 2 longitudinal, and 1 case-control)	Buildings: (i) Quality of housing (thermal and ventilation) (ii) Housing affordability/access to affordable homes or social housing	(i) Mental health (ii) Well-being	No	Quality assessment tool developed by the Effective Public Health Practice Project (EPHPP)
Moore et al.2018 [49]	General population in high-income countries	10 studies (5 longitudinal and 5 cross-sectional)	Built environment: (i) Transport infrastructure modifications (ii) Improving green infrastructure (iii) Urban regeneration	(i) Mental health (ii) Well-being	No	Cochrane risk of bias tool (RoB 2.0) and risk of bias in nonrandomized studies of interventions (ROBINS-I)
Rautio et al.2017 [15]	General population—all ages	57 studies (1 controlled trial, 40 cross-sectional, 9 longitudinal, 1 multicohort, 1 ecologic design, and five cross-sectional and longitudinal)	Living environment: (i) House and built environment (ii) Green spaces (iii) Noise and air pollution	Depression	No	Downs and Black checklist modified by the authors
Turley et al.2013 [50]	Slums—adults/children	1 study (1 controlled study with only postintervention data)	Cement floors (Piso Firme)	(i) Depression (ii) Stress	No	NICE/GATE tool
van den Berg et al.2015 [51]	General population—adults	19 studies (15 cross-sectional and 4 longitudinal)	Green spaces: (i) Amount of green space around the residence in circular buffer (ii) Amount of green space in small area/neighborhood (iii) Presence/number of green spaces within distance (iv) Having a garden (v) Distance to nearest green space (vi) Amount of green space around the residence in circular buffer	Mental health	No	Methodological quality criteria list
Zhang et al. 2017 [52]	People with mobility impairments	12 studies (2 cross-sectional analytical, 2 randomized controlled trials, 1 quantitative descriptive study, 1 nonrandomized controlled trial, 4 phenomenology, and 2 qualitative description)	Health-promoting nature access: (i) Surrounding nature of nursing homes (ii) Green environment near retirement homes (iii) Outdoor blue and green spaces	Mental health	No	Mixed Methods Appraisal Tool (MMAT)

^*∗*^Articles included in the RS with the outcome mental health.

**Table 3:** Assessing Methodological Quality of Systematic Reviews-2 (AMSTAR-2) tool for the assessment of multiple systematic reviews and financial sources of support.

Author (year)	Overall confidence	Financial sources of support
Bowler et al. [44]	Critically low	Natural England Contract FST20-84-037 to ASP
Friesinger et al. [43]	Critically low	No report
Gascon 2015 et al. [45]	Critically low	CERCA Institutes Integration Program (SUMA 2013)
Gascon 2017 et al. [46]	Critically low	European Union's Horizon 2020 research and innovation program under grant agreement no. 666773
Gong et al. [47]	Critically low	No report
Ige et al. [48]	Critically low	Wellcome Trust through the Wellcome Trust Sustaining Health Award (Award number: 106857/Z/15/Z)
Moore et al. [49]	High	(MR/KO232331/1) from the British Heart Foundation, Cancer Research UK, Economic and Social Research Council, Medical Research Council, the Welsh Government, and the Wellcome Trust
Rautio et al. [15]	Critically low	Academy of Finland (268336), European Union's Horizon 2020 research and innovation program (under grant agreement no. 633595) for the DynaHEALTH action and by the European Commission (Grant LifeCycle—H2020—733206)
Turley et al. [50]	High	Internal sources: no sources of support supplied
		External sources: International Initiative for Impact Evaluation (3ie), UK; Jawaharlal Nehru Institute of Advanced Study, Jawaharlal Nehru University, India; Victorian Health Promotion Foundation (VicHealth), Australia
van den Berg et al. [51]	Critically low	European Commission as part of the7th Framework project “Positive health effects of natural environment for human health and well-being (PHENOTYPE)” (grant agreement no. 282996)
Zhang et al. [52]	Critically low	Danish Nature Agency, Ministry of Environment and Food of Denmark (grant number: NST-843.00021), the Bevica Foundation (grant number: 2015–7018), 15. Juni Fonden (grant number: 2015-A-66), and the Danish Outdoor Council (grant number: 104052)

**Table 4:** Conclusions according to the outcome reported by authors.

Outcomes	Conclusions reported by authors					
	Unclear	No effect	Probably harmful	Harmful	Probably beneficial	Beneficial
Mental health and well-being	Gascon 2015 et al. [45]	Moore et al. [49]	NA	NA	Gascon 2017 et al. [46]	Bowler et al. [44]
					Ige et al. [48]	
					van den Berg et al. [51]	
					Zhang et al. [52]	
					Friesinger et al. [43]	

Depression and stress	Turley et al. [50]	NA	NA	NA	Rautio et al. [15]	NA

Psychological distress	NA	NA	NA	NA	Gong et al. [47]	NA

## Additional Points

*Differences between Protocol and Review*. We changed the title registered in PROSPERO: Environmental and architectural interventions and their effects on mental health: a systematic review of reviews to Overview of “systematic reviews” of the built environment's effects on mental health. To further increase the coverage of the literature searches, one additional bibliographic database was searched for the review—PsycINFO. We update the search (November 2019). Additionally, while it is common to encounter an overlap of primary studies when producing an overview of systematic review, we calculated the “corrected covered area” (CCA).

## Supplementary Materials

Supplementary Materials Supplementary Material file includes Tables S1 (PRISMA 2009 checklist) and S2 (Search strategy).
