# Supplementary material for: Overview of “Systematic Reviews” of the Built Environment's Effects on Mental Health
Source: J Environ Public Health. 2020 Mar 19;2020:9523127. doi: 10.1155/2020/9523127 (PMC7106933; doi:10.1155/2020/9523127)
Supplement: Supplementary Materials — Supplementary Material file includes Tables S1 (PRISMA 2009 checklist) and S2 (Search strategy). [file 9523127.f1.zip › 9523127.f1/Supplementary material S2.docx]

S2. Search strategy

1. **COCHRANE LIBRARY**

1 Urban Planning.mp.

2 Town Planning.mp.

3 Urban Renewal.mp.

4 Urban Development.mp.

5 Blue spaces.mp.

6 Architecture.mp.

7 Built environment.mp.

8 Controlled Environment.mp.

9 Urban green spaces.mp.

10 Dwellings.mp.

11 mental health.mp.

12 Mental Disorders.mp.

13 Anxiety Disorders.mp.

14 (Bipolar and Related Disorders).mp.

15 (Disruptive, Impulse Control, and Conduct Disorders).mp.

16 Anxiety, Separation.mp

17 City planning.mp.

18 Green spaces.mp.

19 1 or 2 or 3 or 4 or 5 or 6 or 7 or 8 or 9 or 10 or 17 or 18

20 11 or 12 or 13 or 14 or 15 or 16

21 systematic review.mp. [mp=title, abstract, full text, keywords, caption text

22 meta-analysis.mp. [mp=title, abstract, full text, keywords, caption text]

23 overview.mp. [mp=title, abstract, full text, keywords, caption text]

24 21 or 22 or 23

25 19 and 20 and 24

1. **MEDLINE-EMBASE**

1 City planning.mp.

2 Urban Planning.mp.

3 Town Planning.mp.

4 Urban Renewal.mp.

5 Urban Development.mp.

6 Green spaces.mp.

7 Blue spaces.mp.

8 Architecture.mp.

9 Built environment.mp.

10 Controlled Environment.mp.

11 Urban green spaces.mp.

12 Dwellings.mp.

13 1 or 2 or 3 or 4 or 5 or 6 or 7 or 8 or 9 or 10 or 11 or 12

14 mental health.mp.

15 Mental Disorders.mp.

16 Anxiety Disorders.mp.

17 (Bipolar and Related Disorders).mp.

18 (Disruptive, Impulse Control, and Conduct Disorders).mp.

19 Anxiety, Separation.mp.

20 14 or 15 or 16 or 17 or 18 or 19

21 systematic review.mp. [mp=ti, ab, hw, tn, ot, dm, mf, dv, kw, fx, dq, nm, kf, ox, px, rx, ui, sy]

22 meta-analysis.mp. [mp=ti, ab, hw, tn, ot, dm, mf, dv, kw, fx, dq, nm, kf, ox, px, rx, ui, sy]

23 overview.mp. [mp=ti, ab, hw, tn, ot, dm, mf, dv, kw, fx, dq, nm, kf, ox, px, rx, ui, sy]

24 21 or 22 or 23

25 13 and 20 and 24

1. **PsycINFO**

( City planning OR Urban Planning OR Town Planning OR Urban Renewal OR Urban Development OR Green spaces OR Blue spaces OR Architecture OR Built environment OR Controlled Environment OR Urban green spaces OR Dwellings ) AND ( mental health OR Mental Disorders OR Anxiety Disorders OR ( Bipolar and Related Disorders ) OR ( Disruptive, Impulse Control, and Conduct Disorders ) OR Anxiety, Separation ) AND ( systematic review OR meta-analysis OR overview )

1. **LILACS**

(tw:(City planning )) OR (tw:(Urban Planning )) OR (tw:(Town Planning )) OR (tw:(Urban Renewal )) OR (tw:(Urban Development)) OR (tw:(Green spaces )) AND (tw:(Blue spaces ))OR (tw:(Architecture)) OR (tw:(Built environment )) OR (tw:(Controlled Environment )) OR (tw:(Urban green spaces )) OR(tw:(Dwellings)) AND (tw:( (tw:(mental health )) OR (tw:(Bipolar and Related Disorders )) OR (tw:(Mental Disorders )) OR (tw:(Anxiety Disorders ))
